# The Role of Coronavirus RNA-Processing Enzymes in Innate Immune Evasion

**DOI:** 10.3390/life11060571

**Published:** 2021-06-17

**Authors:** Georgia Mandilara, Marianna A. Koutsi, Marios Agelopoulos, Georgios Sourvinos, Apostolos Beloukas, Theodoros Rampias

**Affiliations:** 1 National Reference Centre for Salmonella and Shigella, School of Public Health, University of West Attica, 11521 Athens, Greece; gmandilara@uniwa.gr; 2 Biomedical Research Foundation of the Academy of Athens, Basic Research Center, 11527 Athens, Greece; mkoutsi@bioacademy.gr (M.A.K.); magelo@bioacademy.gr (M.A.); 3 Laboratory of Clinical Virology, School of Medicine, University of Crete, 71500 Heraklion, Greece; sourvino@med.uoc.gr; 4 Department of Biomedical Sciences, University of West Attica, 12243 Athens, Greece; 5 Institute of Infection and Global Health, University of Liverpool, Liverpool L69 7BE, UK

**Keywords:** viral RNA sensing, coronavirus, innate immunity, immune evasion, SARS-CoV-2

## Abstract

Viral RNA sensing triggers innate antiviral responses in humans by stimulating signaling pathways that include crucial antiviral genes such as interferon. RNA viruses have evolved strategies to inhibit or escape these mechanisms. Coronaviruses use multiple enzymes to synthesize, modify, and process their genomic RNA and sub-genomic RNAs. These include Nsp15 and Nsp16, whose respective roles in RNA capping and dsRNA degradation play a crucial role in coronavirus escape from immune surveillance. Evolutionary studies on coronaviruses demonstrate that genome expansion in Nidoviruses was promoted by the emergence of Nsp14-ExoN activity and led to the acquisition of Nsp15- and Nsp16-RNA-processing activities. In this review, we discuss the main RNA-sensing mechanisms in humans as well as recent structural, functional, and evolutionary insights into coronavirus Nsp15 and Nsp16 with a view to potential antiviral strategies.

## 1. Introduction

Viral infections are classified among the most devastating infectious phenomena, and in certain cases are responsible for the generation of severe pathogenic phenotypes that characterize well-known human diseases [1,2]. The recent dramatic example of severe acute respiratory virus 2 (SARS-CoV-2) spreading among human populations and the subsequent generation and establishment of the COVID-19 pandemic [3,4] exemplifies the capacity of RNA viruses to cause a heavy impact on the global health system. In humans, as the first line of defense against RNA viruses, specific proteins have evolved to sense viral RNA and trigger innate immunity in the early infection phase. On the other hand, the coevolution of RNA viruses with humans has led to a wide variety of viral mechanisms that evade this sensing. Here, we aim to provide a comprehensive update on the role of coronavirus Nsp15- and Nsp16-RNA-processing activities in innate immune escape.

## 2. Classification of RNA Viruses

In prokaryotes, most viruses harbor double-stranded (ds) DNA as genome, while single-stranded (ss) DNA viruses are significantly fewer and there is only a limited presence of RNA viruses. On the contrary, viruses with RNA genomes dominate the eukaryotic virome. The International Committee on Taxonomy of Viruses (ICTV) reports 158 RNA virus species compared to 91 DNA virus species that infect humans [5]. Based on the nature of their genome, RNA viruses are classified as positive-sense (+) RNA viruses, double-stranded (ds) RNA viruses, and negative-sense (-) RNA viruses [6]. Positive-sense (+) single-stranded (ss) RNA viruses that are characterized as human pathogens, belong to Enteroviridae (e.g., Poliovirus), Flaviviridae (e.g., Hepatitis C, Zika virus), Picornaviridae (e.g., poliovirus), Caliciviridae (e.g., norovirus), and Coronaviridae (e.g., SARS-CoV-1) virus families. Since very few treatment options exist, infection by (+)ssRNA viruses represents a major health burden that hardly affects modern societies [7].

The members of (+)ssRNA viruses are characterized by small genomes that rarely exceed 30 kb in size and evolve fast due to the relatively high mutation rate during their replication process and the short generation times [8,9,10]. Despite their limited coding potential, these viruses can replicate efficiently in infected host cells utilizing host proteins and membranes. Their genetic variability due to the high mutation rate allows quick adaptation to environmental changes and promotes resistance to antiviral agents and immune escape from antibodies.

Despite the evolutionary divergence within the group of (+)ssRNA viruses, its members share common features in their replication processes within the host cell. Their replication takes place at the cytoplasmic membranes of the infected cells and is characterized by regulated coordination of RNA synthesis that is mediated by the viral replication complex. Besides the RNA-dependent RNA polymerase (RdRp), this protein complex consists of a core of viral enzymes with protease and RNA helicase activities that are common among most (+)ssRNA viruses. The positive-sense single-stranded RNA genome can be directly translated into viral proteins upon release into the cytoplasm of a host cell. After completion of this translation process, viral replication proteins recruit the viral (+)RNA. Intracellular membranes play an essential role in this step as they can form anchor sites for the assembly of viral RNA replication complexes (VRCs). Moreover, host membranes and proteins may protect and concentrate viral RNAs, and also isolate the replication intermediates from templates during the replication steps [11]. In the next step, the synthesized virus RNA-dependent RNA polymerase (RdRp) generates complementary negative-sense RNA (−RNA), that can be used as a template for the synthesis of additional +RNAs. At an early time post-infection, the population of newly synthesized +RNAs enters a new cycle of translation and replication, while at a later time, +RNA is directed to form new infectious virus particles by encapsulation [12,13,14]. The translation to replication switch for +RNAs is quite essential for virus reproduction.

## 3. Sensors for Long Double-Stranded Viral RNA and Innate Immunity

Recognition of virus-derived nucleic acids is among the most important processes of the host cell defense. During the replication of positive single-stranded RNA viruses (+ssRNA), RNA-replication intermediates such as double-stranded RNA molecules (dsRNA) of more than 30-bp length accumulate. Detection of dsRNAs by specific host protein sensors such as RIG-I-like receptors (RLRs), the protein kinase R (PKR), and oligoadenylate synthetases (OASes) represent some of the central mechanisms of interferon (IFN) pathway activation in infected cells [15]. 

One of the best-studied RLRs with binding activity of non-self dsRNA is the RIG-I (retinoic acid-inducible gene-I), a DExD/H box RNA helicase important for the activation of transcription factors such as IRF3 and NF-κB [16]. RIG-I is a critical regulator for the detection and eradication of the replicating viral genomes. Additionally, MDA5 (melanoma differentiation-associated gene 5), encoded by the *IFIH1* gene in humans, belongs to the same family of cytoplasmic RNA sensors. 

Upon dsRNA binding, these proteins activate the mitochondrial membrane localized adaptor MAVS which in turn, recruits multiple factors (TRAF3, TRAF6, TANK) that promote the formation of a MAVS signaling complex. The latter induces the phosphorylation and nuclear translocation of interferon regulatory factor 3 (IRF3) and interferon regulatory factor 7 (IRF7) as well as the activation of NF-κB [17]. This MAVS-mediated signaling leads to transcriptional activation of type I interferons (IFNs-I) and other proinflammatory cytokines and antiviral genes [17,18]. It is considered that short double-stranded RNA (dsRNA) molecules are more efficiently recognized by RIG-I while MDA5 is activated by longer dsRNAs. In consistency with this pattern of recognition, several studies have shown that RIG-I preferentially recognizes dsRNA signatures by Sendai virus (SeV), vesicular stomatitis virus (VSV), influenza A virus (FLUA), and hepatitis C virus (HCV), while encephalomyocarditis virus (EMCV), norovirus, and murine hepatitis virus (MHV) activate MDA5 [19]. 

PKR is a dsRNA-dependent protein kinase and is transcriptionally upregulated by interferon. This protein exists in an auto-repressed monomeric state and dsRNA binding activates its dimerization and its catalytic activity [20]. Phosphorylation of the eukaryotic translational initiation factor eIF2α by activated PKR suppresses cap-dependent translational initiation. Thus, activation of PKR leads to the protein synthesis shutdown and viral replication inhibition [21]. Besides the direct role of activated PKR in protein translation inhibition, several studies link activated PKR with antiviral interferon and apoptotic signaling pathways since PKR affects diverse transcriptional factors such as IRF1, STATs, p53, ATF-3, and NF-κB [22,23,24]. 

The oligoadenylate synthetase family consists of interferon-induced enzymes (OASes) that upon binding to virally produced dsRNA synthesize 2′−5′ phosphodiester–linked oligoadenylates (2–5 An) which in turn activate the endoribonuclease RNase L within infected cells [25]. Activated RNase L cleaves a wide range of viral and cellular RNAs, including rRNA, tRNA, and mRNA with no sequence specificity and thus induces cell death [26].

Eukaryotic cells have evolved complex networks of specialized response systems to numerous extracellular stimuli, leading to the formation of transcriptional complexes across the appropriate regions of the genome. Virus-inducible gene expression is regulated by virus-activated transcription factors (e.g., NF-κB, IRFs), cis-regulatory elements of the genome such as enhancers, and local chromatin structure [27,28]. The binding of virus-activated transcription factors to specific DNA-binding sites within an enhancer can lead to the assembly of multicomponent complexes termed enhanceosomes, that are critical for gene expression regulation (e.g., the prototype IFN-β enhanceosome) [28,29]. Furthermore, the immune master regulators NF-κB and IRF3 cooperate extensively during innate antiviral transcription in human cells, as shown by a comprehensive genome-wide analysis [30]. Thus, upon detection of (ds)RNAs by specific host protein sensors and upregulation of type I interferon levels, the secreted IFN-I (IFN-α and IFN-β) binds to the IFN receptors (IFNARs) on the surface of the infected and neighboring cells in a paracrine or autocrine manner [31,32] thus commuting the signal of virus-infection, by activating the Janus kinase 1 (JAK1) and tyrosine kinase 2 (TYK2), which in turn phosphorylate the signal transducer and activator of transcription proteins 1 and 2 (STAT1 and STAT2) [33]. 

Phosphorylated STAT1 and STAT2 form a heterodimer, which binds IRF9 to form the trimeric complex called IFN-stimulated gene factor 3 (ISGF3) [34]. The nuclear function of ISGF3 includes binding to IFN-I-stimulated response elements (ISREs), which in turn trigger the expression of interferon-stimulated genes (ISGs) that encode proteins with antiviral role [35,36]. The network of ISG-encoded proteins establishes an anti-viral state that leads to the inhibition of viral transcription, translation, and replication as well as to the degradation of the viral genome [37,38].

## 4. Coronavirus Genome Organization and Innate Immunity Escape Profile

Coronaviruses (CoVs) are RNA viruses (order Nidovirales, family Coronaviridae, subfamily Coronavirinae) characterized by significantly long positive-sense single-stranded RNA genome (26–32 kb). Their genome can be translated by different open reading frames (ORFs) and can also be used as a template for replication and transcription. Negative-sense RNA intermediate is generated to serve as the templates for the synthesis of positive-sense genomic RNA (gRNA) as well as numerous subgenomic RNAs (sgRNAs). Upon the release of genomic RNA into the host cell cytoplasm, translation begins in ORF1a and continues in ORF1b after a −1 ribosome frameshift that occurs immediately upstream of the ORF1a stop codon, producing two polypeptides, pp1a and pp1ab. Proteolytic cleavage of these two polyproteins by virally encoded proteases produces fifteen or sixteen non-structural proteins (Nsps) that are involved in genome replication. Most of the remaining one-third of the genome encodes four structural proteins: the transmembrane (M) glycoprotein, the spike (S) glycoprotein, the envelope (E) protein, and the nucleocapsid (N) protein. The expression of the structural proteins is mainly regulated at the level of transcription via the synthesis of a nested set of subgenomic RNAs (sgRNAs).

CoVs are classified into four genera, Alphacoronavirus, Betacoronavirus, Gammacoronavirus, and Deltacoronavirus infecting a plethora of hosts ranging from birds to mammals [39,40]. The first two genera include only mammalian CoVs and specific members such as the alpha-CoVs 229E, NL63 and the beta-CoVs HKU1 and OC43 infect the respiratory tract of humans with low pathogenicity causing asymptomatic infections to mild cold symptoms [41]. 

More recently, MERS-CoV (Middle East respiratory syndrome, MERS), SARS-CoV (severe acute respiratory syndrome, SARS) and SARS-CoV-2 (coronavirus disease 2019, COVID-19) represent emerging Beta-CoVs of mammal origin that are associated with respiratory illness that may lead to severe pneumonia with massive virus replication and inflammation and acute respiratory distress syndrome (ARDS) [42]. Although the overall mortality of MERS and SARS-CoV is much higher, SARS-CoV-2, which emerged in the Wuhan province in China 2019 [43], is fast spreading, causing a pandemic of coronavirus disease.

Innate immune response is important for: (a) Suppressing the early virus replication phase and consequently reducing the virus load; (b) promoting the antigen presentation and natural killer cell functions; and (c) activating the adaptive immune system. The latter includes the induction of antigen-specific T- and B-cell responses and the development of immunological memory [44]. Therefore, virus infection clearance by antiviral T cells and antibodies is facilitated after a strong and efficient induction of the IFN anti-viral pathway. Through the co-evolution with host cells, viruses have developed diverse strategies to overcome anti-viral responses, evading or delaying early host-cell apoptosis long enough to generate a sufficient yield of progeny virus. Coronaviruses, like other effective viruses, can evade innate immune response during the early infection phase as the latter is characterized by low levels of IFN-I expression or ISGs [45,46,47]. Serum analysis of SARS and COVID-19 patients indicated a lack of IFN-I production despite the elevated cytokine and chemokine levels. It is considered that innate immune escape of SARS-CoV and SARS-CoV-2 is the first step for the progression to a severe immunopathogenesis at the later steps of disease which is characterized by an out-of-control response of the immune system that can lead to lung damage [48,49,50,51,52]. 

Importantly, two major arms of the antiviral gene expression program are shaped within the early virus infection phase. The first one is distinguished by the upregulation of type I and III IFNs followed by the activation of ISGs [45,53] and the second involves the NF-κB activation that leads to secreted chemokine-depended recruitment of subsets of leukocytes [45,54]. The balance between the activation of the NF-κB and IFN pathways is considered as an important factor that influences the severity of the phenotype that emerged upon SARS-CoV-2 infection. SARS-CoV-2 has the ability to downregulate ACE2 upon infection [55]. This results in the upregulation of angiotensin II that through its interaction with its receptor ATR1 modulates the expression of cytokines such as IL-6, TNFa, IL-β etc., through the activation of NF-κB [56], its nuclear translocation and genomic distribution/binding across regulatory regions. The activation of NF-κB axis is considered to activate an antiviral response program that involves the secreted chemokine-depended recruitment of subsets of leukocytes [45,54]. Finally, it is well-accepted that the immune system is a dynamic equilibrium and even in the asymptomatic state there are variations between individuals which influence the immunophenotype of the host [57].

## 5. Coronavirus Proteins Inhibiting Host Innate IFN-I Response

Earlier studies on MERS, SARS-CoV, and Mouse Hepatitis Virus (MHV) have demonstrated that both structural and non-structural proteins of these viruses impair IFN responses in multiple ways. For instance, in both SARS-CoV and MHV, Nsp1 inhibits STAT1 phosphorylation and thereby disrupts IFN signaling [58,59]. Similarly, studies in SARS-CoV have demonstrated that Nsp3 inhibits phosphorylation and nuclear translocation of IRF3-inhibiting IFN signaling [60]. Other non-structural proteins of SARS-CoV that have been identified as inhibitors of IFN induction, are the accessory proteins ORF6 and ORF3b. Both proteins can inhibit activation of IRF3 by phosphorylation, while ORF6 also inhibits the nuclear translocation of STAT1 [61]. On the other hand, the structural N protein of MHV and SARS-CoV is implicated in transcriptional suppression of IFN-β production as well as in inhibition of protein kinase R (PKR) and NF-κB function [62]. SARS-CoV-2 also escapes innate immunity at the early infection phase. Experiments in airway epithelial cell lines have demonstrated that SARS-CoV-2 infection is characterized by a delayed IFN-I induction compared to Sendai virus (SeV) [63]. Interestingly, recent studies indicate a conserved function of accessory protein ORF3b as an effective interferon antagonist in evolutionary clades of SARS-CoV and SARS-CoV-2 [64]. Nsp1 also has a conserved role in weakening the interferon response of host cell in both SARS-CoV and SARS-CoV-2 infections. Nsp1 binds the small ribosomal subunit, inhibiting the translation of host mRNAs and promoting their degradation. Notably, specific interaction of Nsp1 with viral mRNA allows the viral protein expression. As a result, the Nsp1-driven translational shutdown of the host transcriptome inhibits the interferon response and promotes immune evasion [65,66,67].

## 6. Replication-Associated Mechanisms that Contribute to Innate Immunity Evasion

Besides the function of specific viral proteins as interferon antagonists, coronaviruses have evolved several strategies to evade innate immunity. First, coronaviruses replicate in the interior of double-membrane vesicles which prevents recognition of dsRNA replication intermediates by host proteins that sense viral RNA structures [68,69,70]. The expression of hydrophobic viral proteins such as the Nsp3 and Nsp4 of CoVs seems to favor the formation of these replication vesicles that protect the viral dsRNA replication intermediates from innate immune sensors of the cytosol [71].

Like many RNA viruses, coronaviruses protect the 5′ end of their RNA genome and subgenomic RNAs from degradation and evade recognition from the host RNA sensor proteins of the innate immune system by a cap structure generated during replication. More specifically, the viral cap structure at the 5′ end of the RNA molecule consists of a N-methylated guanosine triphosphate and a C2′-O-methyl-ribosyladenine. This structure resembles the cap of eukaryotic mRNA. Viral proteins Nsp14 and Nsp16 catalyze the methylation of the cap on the guanine of the GTP and the C2′ hydroxyl group of the following nucleotide, respectively. Both Nsp14 and Nsp16 are S-adenosylmethionine (SAM)-dependent methyltransferases (MTases). Since host sensor proteins of viral RNA recognize the 5′ cap in order to distinguish the host mRNA from viral RNA, this structure protects coronavirus RNA from recognition by Mda5 and thus prevents Mda5-driven interferon upregulation in virus-infected cells [72]. The important role of cap formation for coronavirus life cycle and immune escape was highlighted by the low virulence of MERS and SARS-CoV strains harboring mutant Nsp16 [73,74]. 

**Figure 1:**
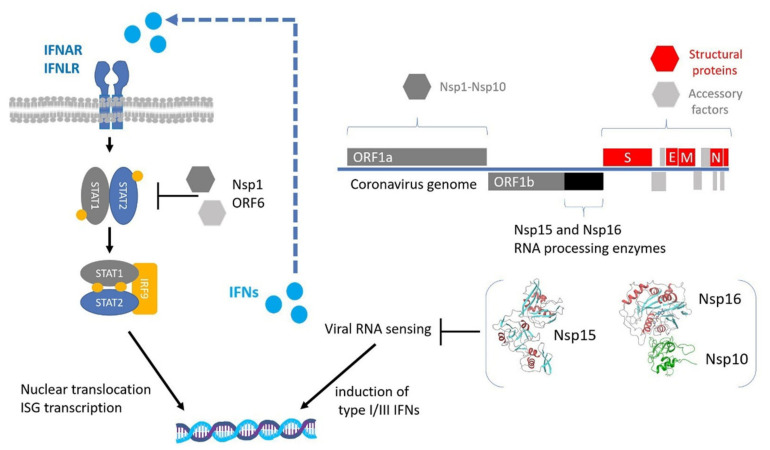
Innate immune evasion by coronaviruses. Upon viral RNA sensing, the expression of type I and III interferons (IFNs) is activated. IFNs are secreted in an autocrine and paracrine manner to induce the expression of interferon-stimulated genes (ISGs) through the STAT1/2 signaling pathway. RNA-processing enzymes Nsp15 and Nsp16 are essential for the escape from viral RNA sensing, while other expressed non-structural or accessory proteins inhibit the STAT1/2 pathway. The regions encoding for the NSP15 and NSP16 proteins are highlighted with black color and their 3D structures are displayed in cartoon representation based on PDB files 6WXC and 6WVN respectively.

Another conserved molecular mechanism associated with innate immune evasion in coronaviruses includes the degradation of dsRNA intermediates by viral proteins with processing activity. Coronavirus encoded Nsp15 protein, a uridine-specific endoribonuclease conserved across coronaviruses is an integral component of the coronaviral replicase-transcriptase complex (RTC) that processes viral RNA to evade detection by host defense systems. This protein is considered a member of the nidoviral EndoU (NendU) family. It is well accepted that Nsp15 uridylate-specific nucleolytic activity on single-stranded and dsRNA limits the formation of dsRNA intermediates and thus inhibits the ability of specific cytoplasmic viral RNA sensors to activate the IFN-I response of innate immunity to infection [75,76]. In this context, loss of Nsp15 nuclease activity in porcine epidemic diarrhea coronavirus (PEDV) leads to the activation of interferon responses and reduced viral titers in infected piglets [77]. Similarly, in vivo experiments with mice infected with MHV strains harboring deficient Nsp15 nuclease, revealed that these infections were associated with attenuated viral replication [78]. Long polyuridine tracts at the 5′-end of negative-strand viral RNA are known to promote host interferon response. The finding that negative-stranded viral RNA intermediates in infected cells with MHV strains harboring catalytically inactive Nsp15, is enriched in polyuridine tracts, further supports the role of Nsp15 in innate immune evasion [75]. The synergism between Nsp15 and Nsp16 and other expressed coronavirus proteins in innate immunity escape is presented in Figure 1.

## 7. Genome Expansion in Coronaviruses: The Evolution of Processes Related to the Protection of 5′ Terminus of RNAs and the Endonucleolytic Cleavage of dsRNA Intermediates

**Figure 2:**
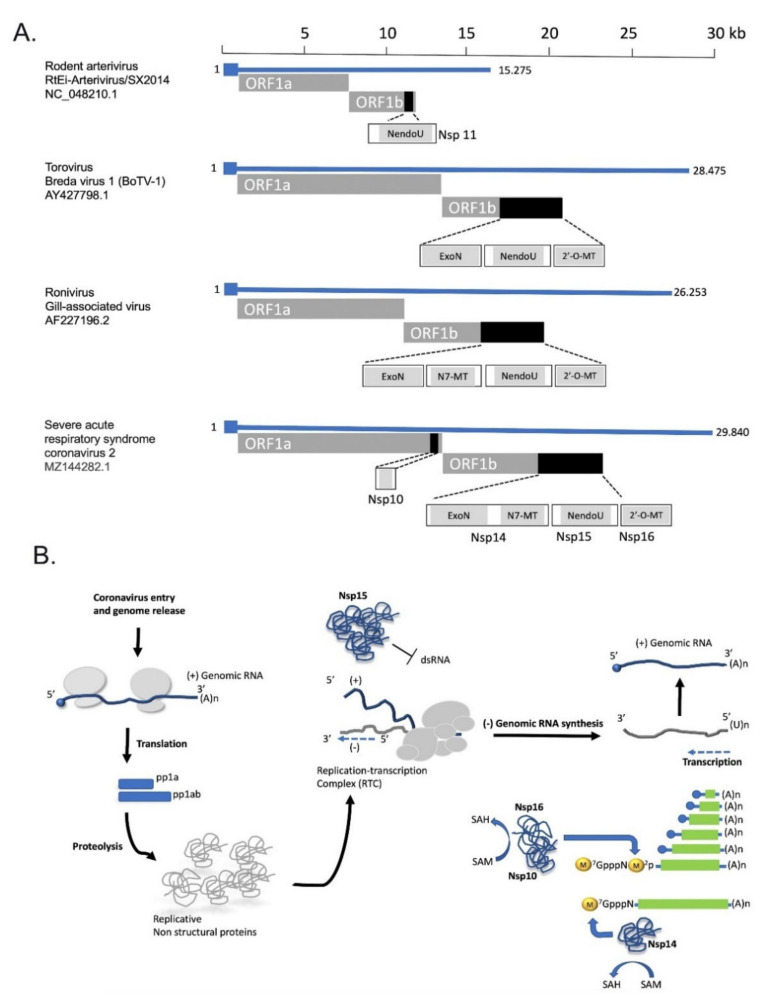
Genome expansion in Nidoviruses and the evolution of viral RNA capping and endonucleolytic cleavage of dsRNA intermediates. (**A**) Genomic organization and expression of ExoN NendoU, N7-MT, and 2′-MT domains in representative members of nidoviruses. (**B**) The functional role of Nsp15 and Nsp16 proteins in coronavirus life cycle. (**A**) n, 3′ polyA sequence; dsRNA, double-stranded RNA; SAM, S-adenosyl methionine SAH, S-adenosyl homocysteine.

The small size of RNA virus genomes has been associated with error-prone replication that lacks proofreading and leads to high mutation rates. Genome expansion in RNA viruses has been identified in order Nidovirales, (Coronaviridae, Arteriviridae, and Roniviridae families) a large group of positive single-stranded RNA viruses that includes those with the largest genomes known to date (larger than 20 kb) that mainly belong to Coronaviridae and Roniviridae families. Those nidoviruses uniquely encode ExoN, an exoribonuclease with RNA 3′ end mismatch excision activity that enhances their replication fidelity and resides in Nsp14 in case of coronaviruses [79]. It is well accepted that ExoN acquisition by nidoviruses enabled genome expansion in Coronaviridae and Roniviridae families [80]. Expansion of the nidovirus genome was accompanied by a gradual acquisition of novel domains (ExoN, NendoU, and O-MT domains), which are directly linked to the evolution of a complex enzymology related to replication, the viral RNA capping and the endonucleolytic cleavage of dsRNA intermediates. The evolution of these processes has facilitated coronaviruses to evade innate immunity (Figure 2). In addition to the function of ExoN as proofreading enzyme in RNA synthesis, its activity is also essential for RNA recombination during coronavirus replication and generation of subgenomic mRNAs. Since coronavirus recombination is a driver for the emergence of novel strains, ExoN activity is considered to contribute to coronavirus variation ([81,82]).

RNA viruses employ diverse methods to add a cap structure or to mimic this structure in the 5′ end of their RNAs in order to protect them from recognition by cytoplasmic sensors. For instance, rhinoviruses that are members of the picornavirus family and harbor a positive single-stranded RNA (+ssRNA) genome, encode a cap mimicking peptide (VPg) to protect the 5′end of their RNA [83]. On the other hand, influenza virus highjacks a short-capped RNA oligonucleotide from host mRNAs in the nucleus during transcription and uses it as an RNA synthesis primer via RNA-dependent RNA polymerase (RdRp). This process is known as «cap snatching» [84]. In the case of negative sense single-stranded (-ss)RNA viruses, such as the respiratory syncytial virus (RSV; Paramyxoviridae family) is able to catalyze the chemical formation of cap-structure in its RNA. Interestingly, recent studies have indicated that the processes of RNA capping and RNA cap methylation are all mediated by RSV large protein (L) which contains three conserved enzymatic domains: the RNA-dependent RNA polymerase (RdRp), the polyribonucleotidyl transferase (PRNTase or capping) domain, and the methyltransferase (MTase) domain, which catalyzes the cap methylation [85,86]. Finally, Togaviridae family viruses, (plus-strand single-stranded RNA viruses) are able to form a 5′ cap structure lacking 2′-O-methylation [87]. Despite the lack of 2′-O-methylation, members of this family, evade innate immune recognition due to specific secondary structures at the 5′ untranslated region of genomic RNA [88].

On the other hand, the NendoU domains that exhibit endoribonuclease activity toward dsRNA intermediates have been identified only in Nidovirales and no homologs have been found in any other RNA viruses. Therefore, NendoU is considered as genetic marker for this order of viruses that discriminates nidoviruses from all other RNA virus families [89]. These ribonucleases have been assigned to Nsp15 and Nsp11 in coronaviruses and arteriviruses respectively. Distant homologs of NendoU have been identified in some prokaryotes and eukaryotes that form a small protein family of endoribonucleases established initially by the Xenopus laevis homolog (XendoU) primarily involved in ribosomal preRNA processing [90].

## 8. RNA Capping in Coronaviruses: Structural and Evolutionary Aspects of CoV Nsp14, and Nsp16 Proteins

In most eukaryotic and viral mRNAs the RNA cap is made of an N7-methylated guanine nucleotide connected through a 5′-5′ triphosphate bridge to the first transcribed nucleotide, generally an adenine. This cap structure formation is typically processed in three sequential steps: (1) An RNA triphosphatase (RTPase) hydrolyses the 5′ γ-phosphate of RNA to generate a 5′ diphosphate RNA end; (2) a guanylyltransferase (GTase), transfers a guanine monophosphate nucleoside (GMP) to the 5′-diphosphate mRNA; and (3) an S-adenosylmethionine (SAM)-dependent (N7-guanine)-methyltransferase (N7MTase) methylates the cap onto the N7-guanine, releasing S-adenosylhomocysteine (SAH). This first methylation in N7, forms a cap-0 structure and is required for the subsequent methylation at the 2′-OH position of the following nucleotide (cap-1 structure) by a SAM-dependent (nucleoside-2′-O-)-methyltransferase (2′-O-MTase). SAM-dependent MTases are present in all life forms, catalyzing the transfer of the SAM methyl group to a wide spectrum of methyl acceptors. Studies of viral MTases involved in RNA capping show low levels of sequence identity and structural similarity. In coronaviruses, the carboxy-terminal part of Nsp14 contains the N7-MTase activity that is required for the addition of a methyl group to the cap guanosine at the N7 position (m7G). The second methylation step at the O-2′ position is associated with the Nsp16 protein (2O-MTase), which requires a cofactor, nsp10, for its proper activity.

CoV Nsp14 is a 60-kDa protein that participates in the formation of the replication–transcription complex. It is considered as a bifunctional enzyme that harbors both 3′-5′exoribonuclease (ExoN) and N7-MTase activities[79,91]. ExoN activity that is located at the amino-terminal part is associated with RNA proofreading during CoV replication. In this context, loss of function mutations in the ExoN active core, has been reported to result in 15- to 20-fold increase in replication errors in (MHV) and SARS-CoV [92,93]. ExoN activity is associated with the evolution of larger viral genomes. It is characteristic that 3′-5′ ExoN activity is found in all large-genome nidoviruses (CoVs, toroviruses, roniviruses, and mesoniviruses)[94]. As mentioned above, the N7-MTase domain of CoV Nsp14 is fused to ExoN domain. Such combination of two different functional domains represents a novel class of RNA-processing proteins in the evolution of Nidoviruses. Moreover, the N7-MTase domain of CoV Nsp14 seems to be unique in the evolution of RNA viruses as its structural analysis revealed a significant structural deviation from the Rrmj fold which is the canonical reference folding for RNA cap MTases [95].

In 2003, the CoV 2′-O-MTase domain was associated to Nsp16 protein[96]. All RNA virus-encoded O-MTases belong to RrmJ/fibrillarin superfamily of ribose 2′-O-methyltransferases, a large group of proteins that modifies 2′-hydroxyl groups of ribose in mRNA, rRNA, and tRNA [97]. This superfamily was prototyped by the heat shock protein RrmJ which is highly conserved from eubacteria to eukarya, and responsible for the 2′-O-ribose methylation of the conserved base U2552 in the A-loop of the 23 S rRNA[98]. Despite the sequence divergence of 2′-O-MTases these enzymes share a conserved pattern of catalytic residues (KDKE catalytic tetrad) and a conserved folding (canonical SAM-MT fold) as defined initially for the O-MTase RrmJ (also named FtsJ), the canonical reference folding for RNA cap MTases[95]. Interestingly, functional experiments on SARS-CoV revealed that Nsp16 alone is inactive and its binding with the small regulatory protein Nsp10 is required to bind both the methyl group donor and the RNA. Moreover, the Nsp16-Nsp10 complex is catalytically active only when the cap guanine is methylated at its N7 position [99,100].

The crystal structure of SARS-CoV and SARS-CoV-2 RNA Cap 2′-O-methyltransferase Nsp16/Nsp10 complex, revealed that Nsp16 from both coronaviruses adopt the canonical O-MTase fold. Moreover, the comparative analysis of their structures showed a high degree of overall fold similarity and a high conservation of residues that form the SAM-binding site [101,102].

## 9. Cleavage of dsRNA Intermediates: Structural and Evolutionary Aspects of CoV Nsp15 Protein

NendoU endoribonuclease activity has been functionally assigned to Nsp15 protein among different human (HCoV-229E, SARS-CoV, and SARS-CoV-2) and animal coronaviruses (MHV) [103,104,105]. Biochemical characterization of the recombinant Nsp15 has indicated that it specifically recognizes uridine moiety and cleaves RNA substrates at the 3′ of target polyuridine tracks through the formation of a 2′-3′ cyclic phosphodiester product [106]. Experiments with bacterially expressed forms of NendoU of severe acute respiratory syndrome coronavirus and HCoV-229E showed that Nsp15 is able to cleave single-stranded RNA but the preferred substrate is double-stranded RNA (dsRNA). Notably, the endonucleolytic activity of Nsp15 is strictly dependent on Mn2+ metal ions [89]. During the coronavirus replication process, Nsp15 cleaves the 5′-polyuridine tracts in (-)-sense viral RNAs that are specifically recognized by the host dsRNA sensor MDA5, impairing innate immune responses [75]. There is a high degree of Nsp15 sequence similarity among coronaviruses. The SARS-CoV-2 Nsp15 shares 88% sequence identity with its known closest homolog from SARS-CoV, 50% sequence identity with the homolog from MERS, and 43% identity with the HCoV-229E homolog. Among Nsp15 endoribonucleases, crystal structures from MHV, SARS, MERS, and HCoV-229E have been reported [107,108,109,110,111]. According to these structural studies, the 39 kDa protein folds into three domains: N-terminal, middle domain, and the C-terminal catalytic NendoU domain. Notably, all these studies support a hexamer model made of dimers of trimers. Specific secondary structure elements of the Nsp15 catalytic domain are conserved in the distant homolog, XendoU, providing further evidence that belong to a common endoribonuclease family [90]. However, Nsp15 structure does not share structural similarities with other characterized ribonucleases, suggesting that this protein adopts a novel folding [108]. Recently, the crystal structure of SARS-CoV-2 Nsp15 demonstrated that SARS-CoV-2 Nsp15 folding is similar to the SARS-CoV, H-CoV-229E, and MERS-CoV homologs [105,112]. The catalytic function of Nsp15 resides in the C-terminal NendoU domain. Within this domain, the main chain architecture and the key catalytic residues His235, His250, Lys290, Thr341, Tyr343, and Ser294 (based on SARS-CoV-2 PDB 6VWW) of the active site are conserved among SARS-CoV-2, SARS-CoV, and MERS-CoV homolog structures. The residues His235, His250, and Lys290 have been proposed to form the catalytic triad, based on the superimposition analysis with the catalytic center of bovine RNase A (His12, His119, and Lys41 based on PDB 5OGH_A). Although the catalytic mechanism of NendoU has not been analyzed in detail, the conservation of the catalytic triad suggests that it may be similar to that of RNase A. In this context, both histidine residues (His235 and His250) act as a general base and general acid in order to promote cleavage. This is also supported by the generation of 2′-3′ cyclic phosphodiester products [113]. However, it is worth noting that the important role of Mn^2+^ ions for SARS-CoV-2 Nsp15 catalysis is conflicting with the proposed RNase A-like reaction mechanism since RNaseA catalysis is metal independent. 

## 10. Drugs Targeting Nsp15 and Nsp16 Proteins

**Figure 3:**
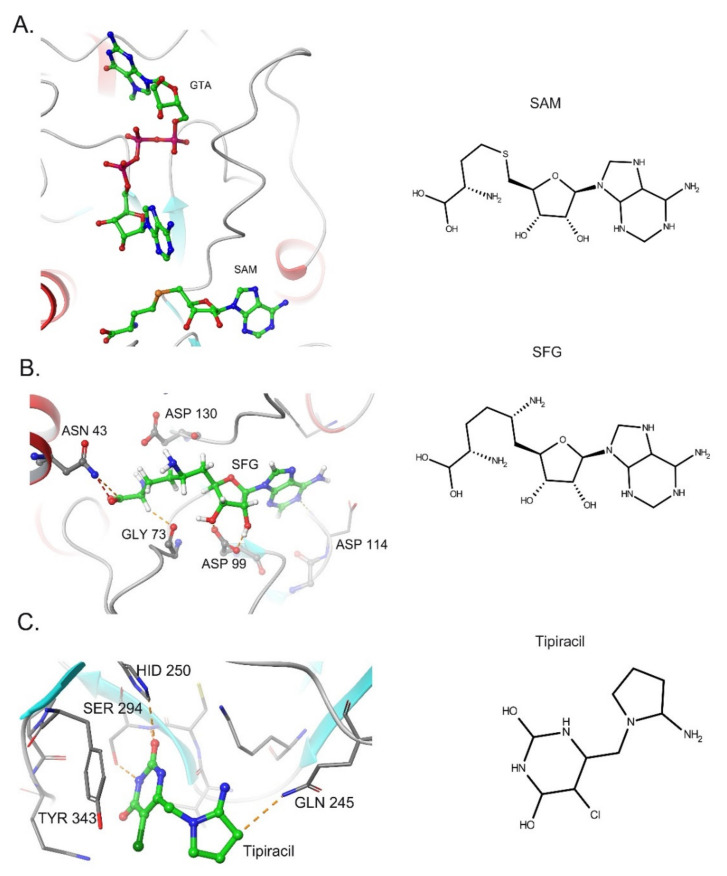
Inhibitors of SARS-CoV-2 Nsp15 and Nsp16 proteins. (**A**). Structure of Nsp16 in Complex with 7-methyl-GpppA (GTA) and S-Adenosylmethionine (PDB: 6WVN). (**B**) Binding of Sinefungin (SFG) in SARS-Cov-2 NSP16 (PDB: 6WKQ). (**C**). Structure of NSP15 Endoribonuclease from SARS CoV-2 in the Complex with drug Tipiracil (PDB: 6WXC).

As emphasized above, Nsp15 and Nsp16 activity are important for SARS-CoV-2 innate immune escape during the viral cycle. Thus, the development of molecules capable of inhibiting these proteins might open new treatment avenues to restore viral RNA recognition and stimulate the host antiviral response against SARS-CoV-2. Recent studies have shown that Nsp16 2′ O-MTase activity can be suppressed by conventional SAM antagonists. For instance, sinefungin (SFG)—a SAM analog—efficiently binds the SAM-binding site of SARS-CoV-2 Nsp16, inhibiting its activity [101] (Figure 3A,B). Crystallographic and biochemical studies have indicated that Nsp10 promotes the stabilization of the Nsp16 SAM-binding pocket and favors the extension of Nsp16 RNA-binding groove[100,101]. Therefore, molecules that can disrupt the Nsp10-Nsp16 interaction or inhibit complex formation are considered putative candidate antivirals. In this context, peptides derived from the conserved interaction between Nsp10 and Nsp16 exhibit an inhibitory effect on 2′-O-MTase activity in vitro [114].

The analysis of Nsp16:Nsp10 crystallographic data for MERS, SARS-CoV, and SARS-CoV-2 have indicated that RNA guanosine cap binds to a region adjacent to the SAM-binding pocket, placing its ribose ring in close proximity to the amino group of SAM [100,101,115]. The binding affinity of different nucleoside/cap analogs in these pockets has been also investigated in viral 2′-O-MTases as candidate antiviral drugs [116]. Notably the GTP analog ribavirin triphosphate has been reported to suppress dengue virus 2′-O MTase activity [117,118]. The design of SAM analogues that also interacts with this adjacent cap-binding site is a challenge for the generation of more potent and specific inhibitors compared to sinefungin [115,119]. In this direction, several groups have performed virtual screening and molecular docking in order to identify alternative candidate drugs [120,121,122].

Nsp15 protein is an attractive target in the field of drug design as it is exclusively present in nidoviruses with no close human homologs identified. Since structural studies indicate a high degree of similarity between the catalytic sites of CoV Nsp15 and RNase A, and a common catalytic mechanism, small molecule inhibitors of RNase A were among the first drugs tested for their ability to inhibit Nsp15 activity. In this direction, Benzopurpurin B and Congo red were shown to display the higher inhibition on Nsp15 activity. In addition, these drugs reduced the infectivity of the SARS-CoV in Vero cells [123]. Based on this evidence, a more comprehensive investigation is needed for the exploitation of RNAse A inhibitors as drugs that can target Nsp15 activity. 

Using the available crystallographic data for NSP15 from MERS, SARS-CoV, and SARS-CoV-2, several groups have employed virtual screening, molecular docking, and molecular dynamic simulation techniques to identify putative antivirals against Nsp15 [123,124]. Nsp15 is a uridine-specific endoribonuclease and amino acid residue Ser294 has been proposed to play a crucial role in uracil recognition in the catalytic pocket [75]. Therefore, synthetic uracil competitors may represent a promising group of small molecules for drug development against Nsp15. Notably, the uracil analog Tipiracil has been shown to bind the uracil site in the catalytic pocket of SARS-CoV-2 Nsp15 as a competitive inhibitor and suppresses its activity in vitro experiments [125] (Figure 3B). 

## 11. Conclusions

RNA viral recognition by cytosolic RNA sensors can stimulate the type I and III IFNs production that in turn, establish the cellular antiviral response and activate the adaptive immunity. The co-evolution of RNA viruses with their host has led to the emergence of viral evasion mechanisms of intracellular RNA sensing. Upon early phases of coronavirus infection, IFN response appears to be reduced and delayed indicating an innate immune escape. At the molecular level, the cap structure formation to the 5′ end of the viral genome and subgenomic transcripts and the degradation of dsRNA intermediates that are produced during replication is considered to hide coronaviruses from host sensor proteins that are able to stimulate the IFN signaling pathway. In the current review, the specific function of coronavirus-encoded enzymes Nsp15 and Nsp16 on dsRNA degradation and viral cap methylation respectively, have been discussed. These RNA-processing proteins and the associated innate immune escape mechanisms have evolved in nidovirales after the acquisition of the proofreading exoribonuclease (ExoN) activity and the expansion of their genomes. This suggests that the specific innate immune escape profile of coronaviruses has been developed in close association with complex evolutionary processes related to gain of Nsp14 proofreading activity, and genome expansion events. Progress in the field of coronavirus genome replication biology will allow us to understand how evolution can lead to the acquisition of new RNA enzymatic activities, which in turn provides the capability to modulate the innate immune response. It is characteristic that Nsp15 is exclusively present only in nidovirales evolutionary clade. Since coronaviruses use host proteins as part of their replication processes, it has also become clear that Nsp15 and Nsp16 protein evolution and function were affected by virus–host interactions. For instance, it has been proposed that NSP15 degrades the excess amount of dsRNA that escapes from replication-transcription complexes in the inner core of cytoplasmic vesicles that are formed by host membranes and proteins. Since the innate immune escape mechanisms are based on complex virus–host interactions, it is possible that Nsp15 and Nsp16 RNA-processing enzymes have evolved to synergize with other viral Nsp proteins that function as IFN antagonists to counteract anti-viral immune responses.

Numerous studies have highlighted the importance of bypassing or modulating innate immune defense for coronavirus replication. A key challenge is to translate this knowledge into useful applications for the development of new antivirals. In this direction, the pharmacological inhibition of Nsp15 and Nsp16 proteins may potently induce antiviral responses for long-lasting immunity.

## Data Availability

Not applicable.
